# Swab Sample Transfer for Point-Of-Care Diagnostics: Characterization of Swab Types and Manual Agitation Methods

**DOI:** 10.1371/journal.pone.0105786

**Published:** 2014-09-02

**Authors:** Nuttada Panpradist, Bhushan J. Toley, Xiaohong Zhang, Samantha Byrnes, Joshua R. Buser, Janet A. Englund, Barry R. Lutz

**Affiliations:** 1 Department of Bioengineering, University of Washington, Seattle, Washington, United States of America; 2 Program in Infectious Diseases, Clinical Research Division, Fred Hutchinson Cancer Research Center, Seattle, Washington, United States of America; 3 Department of Pediatrics, Seattle Children's Research Institute, University of Washington, Seattle, Washington, United States of America; Rockefeller University, United States of America

## Abstract

**Background:**

The global need for disease detection and control has increased effort to engineer point-of-care (POC) tests that are simple, robust, affordable, and non-instrumented. In many POC tests, sample collection involves swabbing the site (e.g., nose, skin), agitating the swab in a fluid to release the sample, and transferring the fluid to a device for analysis. Poor performance in sample transfer can reduce sensitivity and reproducibility.

**Methods:**

In this study, we compared bacterial release efficiency of seven swab types using manual-agitation methods typical of POC devices. Transfer efficiency was measured using quantitative PCR (qPCR) for *Staphylococcus aureus* under conditions representing a range of sampling scenarios: 1) spiking low-volume samples onto the swab, 2) submerging the swab in excess-volume samples, and 3) swabbing dried sample from a surface.

**Results:**

Excess-volume samples gave the expected recovery for most swabs (based on tip fluid capacity); a polyurethane swab showed enhanced recovery, suggesting an ability to accumulate organisms during sampling. Dry samples led to recovery of ∼20–30% for all swabs tested, suggesting that swab structure and volume is less important when organisms are applied to the outer swab surface. Low-volume samples led to the widest range of transfer efficiencies between swab types. Rayon swabs (63 µL capacity) performed well for excess-volume samples, but showed poor recovery for low-volume samples. Nylon (100 µL) and polyester swabs (27 µL) showed intermediate recovery for low-volume and excess-volume samples. Polyurethane swabs (16 µL) showed excellent recovery for all sample types. This work demonstrates that swab transfer efficiency can be affected by swab material, structure, and fluid capacity and details of the sample. Results and quantitative analysis methods from this study will assist POC assay developers in selecting appropriate swab types and transfer methods.

## Introduction

Diagnostics for non-blood-associated pathogens often use swabs as a specimen-collecting tool. For example, swabs are used to collect throat specimens for Group A *Streptococcus* [1]; nasal and nasopharyngeal specimens for *Staphylococcus aureus* [2], [3], *Bordetella pertussis*, influenza virus [4], [5], and respiratory syncytial virus (RSV) [6], [7]; female endocervical or male urethral specimens for *Neisseria gonorrhea* [8] and *Chlamydia trachomatis* [9]; and fecal swabs for viral gastroenteritis [10]. Depending on the source of collection, swabs should have shaft properties (flexibility, length) and tip size/shape appropriate for the sampling site, and the swab tip material and microstructure should provide efficient sample capture and target release in the presence of sample matrix components (e.g., human cells, body fluids, and other contaminants). Commercially available swabs are currently utilized with a variety of swab tip materials (e.g., nylon, rayon, cotton, polyester, polyurethane, and alginate polymer) and microstructures (e.g., tightly wound, knitted, flocked fiber, and reticulated). In laboratory settings, swabs are typically agitated by vortex mixing to release organisms into a transfer fluid [11]–[15] that is analyzed by culture, immunoassays (ELISA), or nucleic acid tests (PCR).

Swab sampling and fluid transfer are also used in lateral flow tests (LFTs) intended for point-of-care (POC) testing in non-laboratory settings. Commercial LFTs that are being used with swabs worldwide include rapid streptococcal antigen assays, respiratory syncytial virus (RSV) assays: BinaxNOW RSV Lateral Flow (Alere Inc., Waltham, MA), RSV Respi-Strip (Coris Bioconcept, Namur, Belgium); and influenza detection tests: BinaxNOW Influenza A&B Card (Alere Inc., Waltham, MA), QuickVue Influenza Test (Quidel Corp., San Diego, CA). Typical instructions require the user to dip the swab into transfer fluid (∼0.7 mL to 1 mL), manually agitate the swab for a specified time [16], and transfer a fraction of the fluid (∼100 µL) to the device [17]. The low fluid capacity of LFTs results in most of the sample being discarded, and manual agitation may be less effective than vigorous vortex mixing used in laboratory settings. Since the sensitivity of LFTs is typically lower than laboratory-based tests, there is a need to maximize transfer of sample from the swab to the device.

Studies evaluating swab transfer use widely varied definitions of transfer efficiency, often focus on a specific clinical application, and typically rely on qualitative analysis techniques. Thus, we built on previous work to develop quantitative analysis methods and definitions for swab transfer efficiency that can be applied to a variety of swab types, sampling applications, and transfer methods. We present methods to quantify swab transfer efficiency, discuss potential pitfalls that could bias quantitative analysis, and evaluate transfer efficiency for a range of swab types, sample properties, and manual agitation methods that meet the unique needs for POC applications. The results and discussion can aid researchers or test developers in rational selection of swabs and transfer methods for diagnostics development in both POC and laboratory settings.

## Materials and Methods

### Model organism (Staphylococcus aureus)

A single colony of *S. aureus* (ATCC 25923) was inoculated in tryptic soy broth (TSB) (Fisher B11768) and shaken overnight (250 rpm, 37°C). The overnight culture was further diluted 1∶100 in TSB and incubated (250 , 37°C) for 3 hours until the OD_600_ reached 2±0.2, corresponding to a concentration of ∼10^9^ CFU/mL. *S. aureus* bacteria were then harvested and resuspended in one of two buffers: 1X Tris-EDTA buffer at pH 8.0 (TE: 10 mM Tris-HCl +1 mM EDTA) or TE buffer with in-house human simulated nasal matrix (SNM: 110 mM NaCl, 1% w/v mucin from porcine stomach Type III (Sigma, M1778-10G) and 10 µg/mL w/v human genomic DNA (Promega G3041)) at 90% v/v of TE/SNM.

### Swabs and agitation methods

Seven different commercially-available swabs were tested: rayon (Copan Diagnostics Inc., 170KS0, Murrieta, CA), cotton (Puritan Medical Products Co., LLC, 25–806, Guilford, ME), mid-turbinate (MT) flocked nylon (Copan Diagnostics Inc., 56380CS01, Murrieta, CA), regular-tip flocked nylon (Copan Diagnostics Inc., 502CS01, Murrieta, CA), polyester (PES) (Contec Inc., 19059209, Spartanburg, SC), polyurethane (PUR) (Foamtec International, 19304613, Oceanside, CA), and calcium alginate (Puritan Medical Products Co., LLC, 25-806-2PA, Guilford, ME).

Vortexing is commonly used in the laboratory to maximize release of organisms from swabs, but it is likely not available in low resource settings. Agitation methods for POC tests are normally specified in units of time, without definition of the method. For consistency across experiments, we defined a base case method for translating the submerged swab along a circular path against the side of the tube at a rate of 1 cycle per second (1 Hz) for a specified time (e.g., “10 second 1 Hz side twirl”). The swab agitation was done by hand but using the timer as a reference for manual control of the twirling rate. The potential errors introduced by the users were not excluded in the data. The impact of these variations was tested (below in the section titled “*Robustness to user variations in manual agitation”).*

After the sample was applied to swabs, swabs were agitated in 128 µL of transfer fluid. This volume was chosen to be compatible with the fluid capacity of typical POC devices (∼100 µL for LFT) to enable complete utilization of the sample fluid.

#### Volume recovery experiments

To quantify fluid release volume, 15 µL TE buffer was pipetted onto the tip of a dry swab. The swab was then dipped into pre-weighed 128 µL TE buffer (or 1% w/v sodium citrate solution for calcium alginate swab) and agitated (10 second 1 Hz side twirl). The swab was removed, and the fluid left in the tube was weighed. The volume left in tube was calculated (Equation 1) and compared to a control (Equation 2). As the control, 15 µL TE buffer was pipetted into 128 µL of pre-weighed TE buffer, and the fluid in the tube was weighed. The % Volume Recovery was calculated using Equation 3.
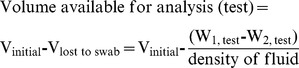
(1)

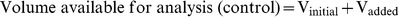
(2)

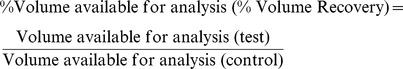
(3)


Where 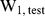  =  initial weight of test tube containing 128 µL TE buffer


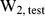
 =  weight of test tube after swab transfer and removal


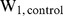
 =  initial weight of control tube containing 128 µL TE buffer


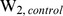
 =  weight of control tube after 15 µL TE buffer was added


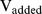
  =  added volume of TE (either onto swab or onto control tube), 15 µL


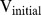
  =  initial volume of TE in test or control tube, 128 µL


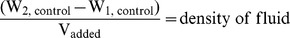
, used to convert weight change of fluid in the test tube 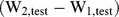 into volume change. 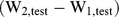 is negative when the swab absorbed more fluid from the tube than it released. Adding
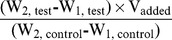
 to 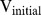 would provide the recovered volume in the test tube (Volume available for analysis (test)).

A transfer volume of 128 µL was selected because this volume allowed at least 20 µL to be recovered after the swab transfer step for all swab types for further testing by qPCR (as described in the next section); it also represents an appropriate volume for LFTs.

#### Organism recovery experiments

Bacteria from swabs and control samples were lysed using a method modified from Patel *et al* [18], and lysed samples were analyzed by quantitative PCR (qPCR). Regular-tip nylon, MT nylon, and rayon swabs were obtained in sterile packaging, and PES and PUR swabs and other materials were autoclaved prior to use. All swabs and materials were tested for bacteria and DNA bacteria contamination. In all experiments, bacteria were transferred from the swab to 128 µL of 3 U/µL achromopeptidase (ACP) (Sigma #A3547) in TE to lyse the bacteria. Lysis continued for 2 minutes at room temperature. Following lysis, ACP was deactivated by heating (10 minutes, 95°C), and the sample tube was placed in ice immediately. Bacterial lysate was filtered through a centrifuge tube filter (0.2 µm pore size) to remove debris. 9 µL of the filtered lysate was analyzed by a MRSA/SA ELITe MGB qPCR assay (Elitech Group Molecular Diagnostics, formerly Epoch Biosciences, #M800346). This assay detects a single open reading frame encoding *S. aureus*-specific lactate-dehydrogenase-1 (*ldh1*). The concentration of *ldh1* copies in each sample (copies/µL) was estimated based on a standard curve (80 to 8×10^8^ *ldh1* copies). To verify that swabs were not contaminated during experiments, negative control samples (15 µL of TE buffer (N = 3) and swabs with no added bacteria (N = 3)) were transferred to 128 µL of lysis buffer and analyzed by the same procedures as other samples.

*S. aureus* was applied to swab tips using one of the three methods described in the following sections and eluted into 128 µL of pre-weighed lysis buffer. In the control case, *S. aureus* was introduced directly to 128 µL of pre-weighed lysis buffer. Both test and control tubes were weighed and set aside to allow for the lysis to complete. Filtered lysate then underwent qPCR for quantitation of *ldh1* concentration (copies/µL). The limit of detection (LoD) of qPCR was less than 5 *ldh1* copies per reaction, corresponding to about 1 CFU per reaction (Fig. S1). Weights of test and control tubes were used to calculate volumes left in the tubes (Equation 1 and Equation 2, respectively). The number of *ldh1* copies left in tube in each case was calculated using Equation 4, and the % Organism Recovery was calculated using Equation 5.
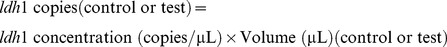
(4)

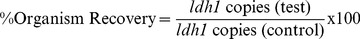
(5)


Further details on how *S. aureus* was introduced to swabs in each method are described below.

*Low-volume fluid sample (less than swab saturation)*

15 µL *S. aureus*/TE (100, 10^4^, and 10^6^ CFU) was pipetted onto a swab tip. The amount of bacteria input exceeded the LoD of the qPCR assay (Fig. S1). The swab was then dipped into lysis buffer, manually agitated (as described above or 10 second 1 Hz side twirl), and removed. The number of *ldh1* copies was measured and compared to a control case in which *S. aureus*/TE solution was directly pipetted into pre-weighed lysis buffer (with no submergence of pipette tip to avoid introducing bacteria from the outside surface of the tip). A fresh pipette tip was then used for mixing.

### Excess-volume fluid sample (beyond swab saturation)

A dry or a pre-wet (by dipping into TE) swab was dipped into 1 mL of *S. aureus*/TE solution (10^6^ CFU/mL) and manually agitated (10 second 1 Hz side twirl) to load sample into the swab. Swabs were transferred into pre-weighed lysis buffer, manually agitated, and removed. The number of *ldh1* copies in the lysis tube was measured and reported.

### Sample dried on a surface

Dried bacterial samples were prepared by pipetting 15 µL of *S. aureus*/TSB (10^4^ CFU) onto sterilized 25/46-inch diameter polydimethylsiloxane (PDMS) punches and left in a desiccator for 30 minutes. PDMS was chosen as the surface since it is known to not adhere to *S. aureus* organisms [19], and we attempted to remove effects of collection efficiency by using a very vigorous swabbing procedure (goal of 100% collection). A dry or a pre-wet swab was rubbed across the PDMS surface 10 times to pick up organisms (Fig. S2), transferred into the pre-weighed lysis buffer (128 µl), manually agitated, and removed. The number of *ldh1* copies in the lysis tube was measured and compared to a control. In the control, the eluate was derived from placing the PDMS punch with dried bacteria directly in lysis buffer and vortexing for 10 seconds.

### Robustness to user variations in manual agitation

15 µL of *S. aureus*/TE solution (10^4^ CFU) was pipetted onto a dry swab, and the swab was dipped into lysis buffer. Different manual-twirling methods were used to release bacteria. “Side twirl” refers moving the swab tip around the interior side of the tube in a circular motion. “Bottom twirl” refers to placing the swab tip at the bottom of the tube and rotating the shaft.

### Engineering for improved recovery

A forced-flow method using a syringe was developed as an alternative to manual agitation to improve organism recovery. In the test case, 15 µL of *S. aureus*/TE (10^4^ CFU) was introduced onto a dry swab, and the swab was dipped into the lysis buffer tube, the bottom of which was connected to a syringe. The plunger of the syringe was then pushed and pulled 5 times. At the end, the swab remained in the tube whereas the eluate was contained within the syringe piston and could be purged through an opening in the side of the syringe.

### Bench-top gold standard method

Swabs (N = 5) were tested for organism recovery using a laboratory protocol: vortexing for 10 seconds at the maximum speed. In the test case, 15 µL of *S. aureus*/TE (10^4^ CFU) was introduced onto a dry swab, and the swab was dipped into lysis buffer prior to vortexing.

### Data Analysis

Statistical analysis was performed using MATLAB (MathWorks, Natick, MA). Analysis of variance (ANOVA) was used to test for significant differences among means. In the case that ANOVA indicated significant differences (p<0.05), post-hoc comparisons (Tukey-Kramer procedure, adjusted for multiple comparisons) were used to determine which means were significantly different from one another. The data and analysis underlying the findings are fully available on request.

## Results

**Figure 1:**
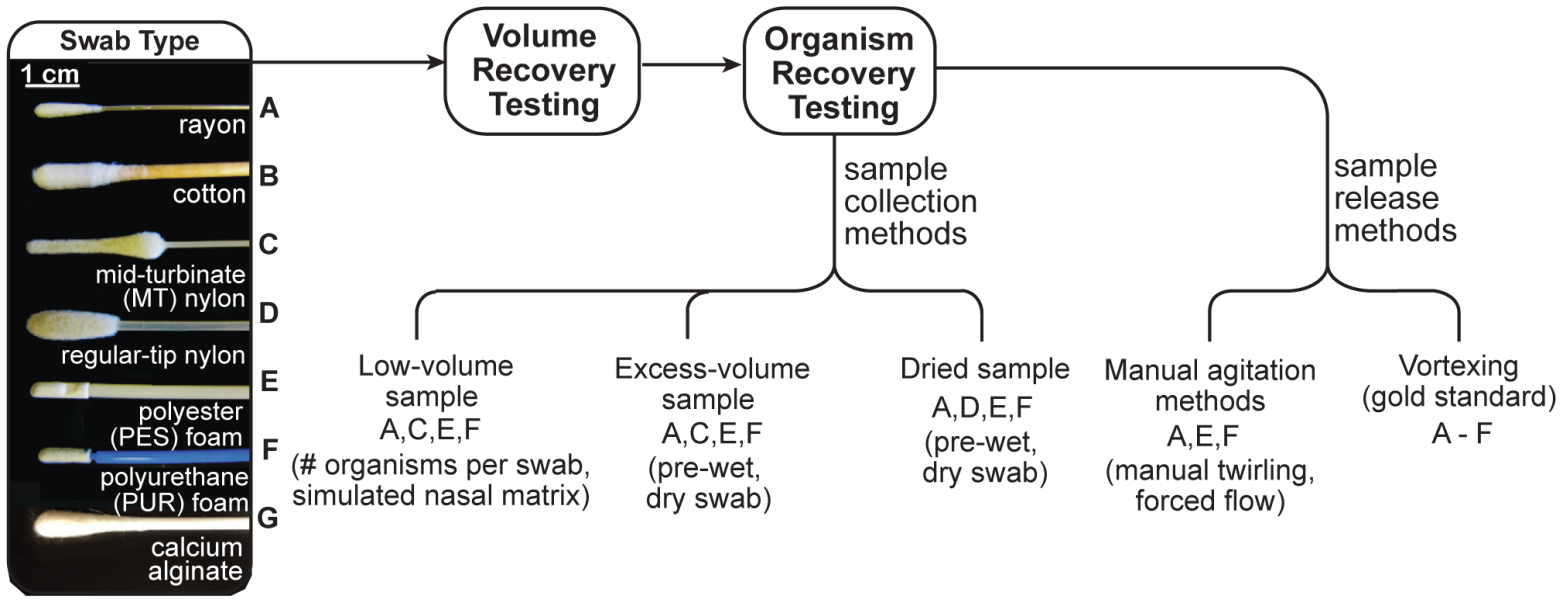
Schematic of swab transfer experiments. Seven commercially-available clinical swabs (labeled A, B, C, D, E, F, and G) were tested for volume recovery and organism recovery. Organisms were applied to swabs in three ways: pipetting a low-volume sample onto the swab, dipping the swab into excess-volume sample, or rubbing the swab across dried sample on a surface. Selected cases included variation in sample concentration, addition of simulated nasal matrix, and comparison of dry and pre-wet swabs. Different manual swab agitation methods, manual twirling and forced flow, were tested for their effects on swab transfer efficiency compared to vortexing (gold standard method).

A variety of swab types were evaluated under different conditions, as illustrated in Figure 1. We selected a set of commercially-available swabs representing a range of materials, microstructure, and size: low absorbent foam (reticulated polyurethane swab – PUR and knitted-pattern PES swabs – PES), low absorbent fiber (MT nylon flocked swabs – MT nylon, and regular-tip nylon flocked swabs), high absorbent fiber (tightly wound cotton and rayon swabs), and dissolvable swabs (calcium alginate swabs). Figure S3 shows scanning electron microscopy images of the nylon (flocked), PUR (reticulated), PES (knitted), and rayon (wound) swab tips; this set exhibits a wide variety of material structures and pore sizes. We focused on the manual “insert and twirl” agitation method common for LFTs and used a reduced fluid transfer volume (∼100 µL) appropriate for typical LFTs. Swabs were evaluated in a series of tests, with subsets of swabs chosen for each test to illustrate key differences and analysis methods. Swabs were first tested for fluid retention (loss of fluid sample to the swab), and swabs with low retention volume were carried forward to test organism recovery. We used *Staphylococcus aureus* as a model system, with recovery quantified by quantitative polymerase chain reaction (qPCR). Organisms were applied to swabs under various conditions to represent a range of sampling conditions from dry to wet: a) low-volume fluid sample (less than swab saturation), b) high-volume fluid sample (beyond swab saturation), and c) sample dried on a surface. For the low-volume sample condition, recovery was tested for a wide range of concentrations (100 to 10^6^ organisms) [20], and a simulated human nasal matrix was used as an example of a complex sample. Pre-wet swabs were compared to dry swabs for the cases of excess sample volume and dry sample collection. We also tested variations on manual agitation to identify sensitivity to user operation, and demonstrated an engineered manual agitation method to improve recovery for swab-sample combinations that performed poorly. The data from all experiments are summarized in Tables S1 and S2.

### Volume recovery

**Figure 2:**
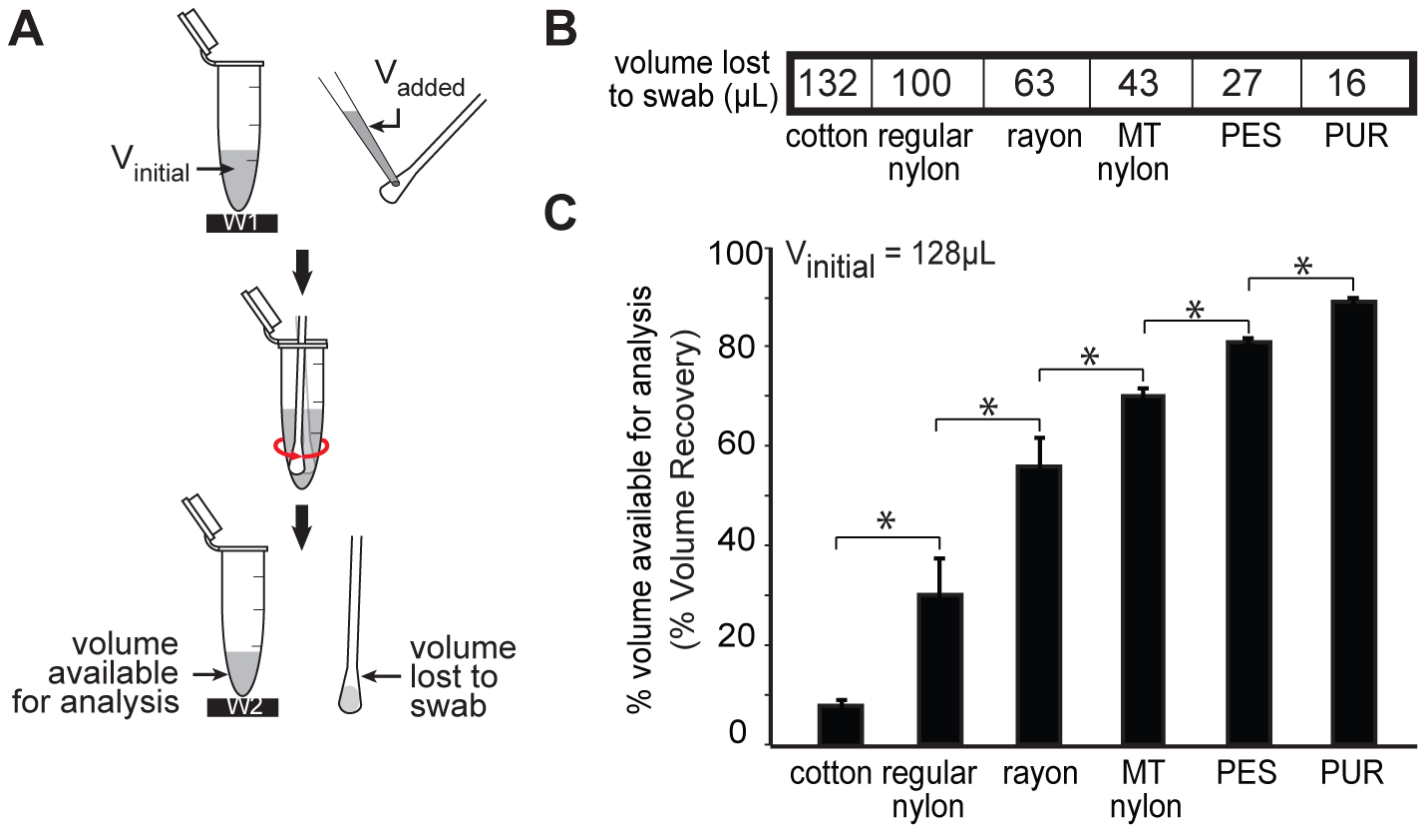
Volume recovery testing. (A) Schematic of the experimental setup. The tube containing 128 µL TE was weighed (W1), and 15 µL TE was pipetted onto the swab, which was then transferred into the tube using 10 second 1 Hz side twirl, and removed. The tube containing the leftover buffer (eluate) was weighed (W2). The % volume available for analysis (% Volume Recovery) was calculated using Equation 3 in the text. (B) Mean TE volume (µL) absorbed by each type of swab (N = 5). (C) Comparison of the % Volume Recovery (mean ± SE; N = 5) from each swab. Calcium alginate swabs were resuspended in 1% w/v sodium citrate buffer to dissolve fibrous tip materials, the % Volume Recovery was not reported here due to density change of the buffer during (A). * indicates significant differences (Tukey-Kramer, α = 0.05).

Fluid volume retention for seven commercially-available swabs is shown in Figure 2. Swabs with 15 µL of added fluid were inserted into tubes containing 128 µL of fluid and removed, and the fluid remaining in the tube was used to calculate fluid volume lost to the swab (Fig. 2A). Figure 2B shows the fluid volume lost to each swab, and Figure 2C shows the percent volume available for analysis (% volume recovery) for a starting volume of 128 µL. PUR and PES yielded the highest volume recovery (PUR: mean ± SE  = 89±0.4%; PES: 81%±0.3%). Conversely, cotton and regular-tip nylon swabs retained more fluid resulting in the poorest volume recovery (cotton: 8±0.6%; regular-tip nylon: 30±3.4%). The remaining swabs, rayon and MT nylon, had intermediate volume recovery (rayon: 56±2.5%; MT nylon: 70±0.8%). All data points in Figure 2B and 2C have 5 replicates. A one-way ANOVA indicated that volume recovery differed significantly across swab types (p<0.0001). Post-hoc comparisons indicated that PUR had a significantly higher volume recovery than other swab types (p<0.05). Additionally, we tested dissolvable calcium alginate swabs in the recommended dissolution buffer (sodium citrate). The recommended procedure uses a buffer volume that is too large for LFTs (15 mL), and using the small volume (128 µL) resulted in a glue-like gel that would not flow through an LFT (Fig. S4).

### Organism recovery

We determined that our modified ACP lysis method gave the same amount of amplifiable DNA as the original method by Patel *et al* (Fig. S5) [18]. Eluate recovered from swabs was devoid of bacteria or bacterial DNA (Fig. S6) and did not interfere with qPCR or ACP lysis (Fig. S7). The qPCR assay reported 3–6 genomic copies per CFU across all experiments; along with each experiment result we report the sample CFU for reference, but recovery values reported in this paper are based on copies measured by qPCR. Results are reported as absolute organisms recovered (Eqn. 4) or as % organism recovery based on a control sample analyzed by the same method (Eqn. 5).

#### Low-volume fluid samples

**Figure 3:**
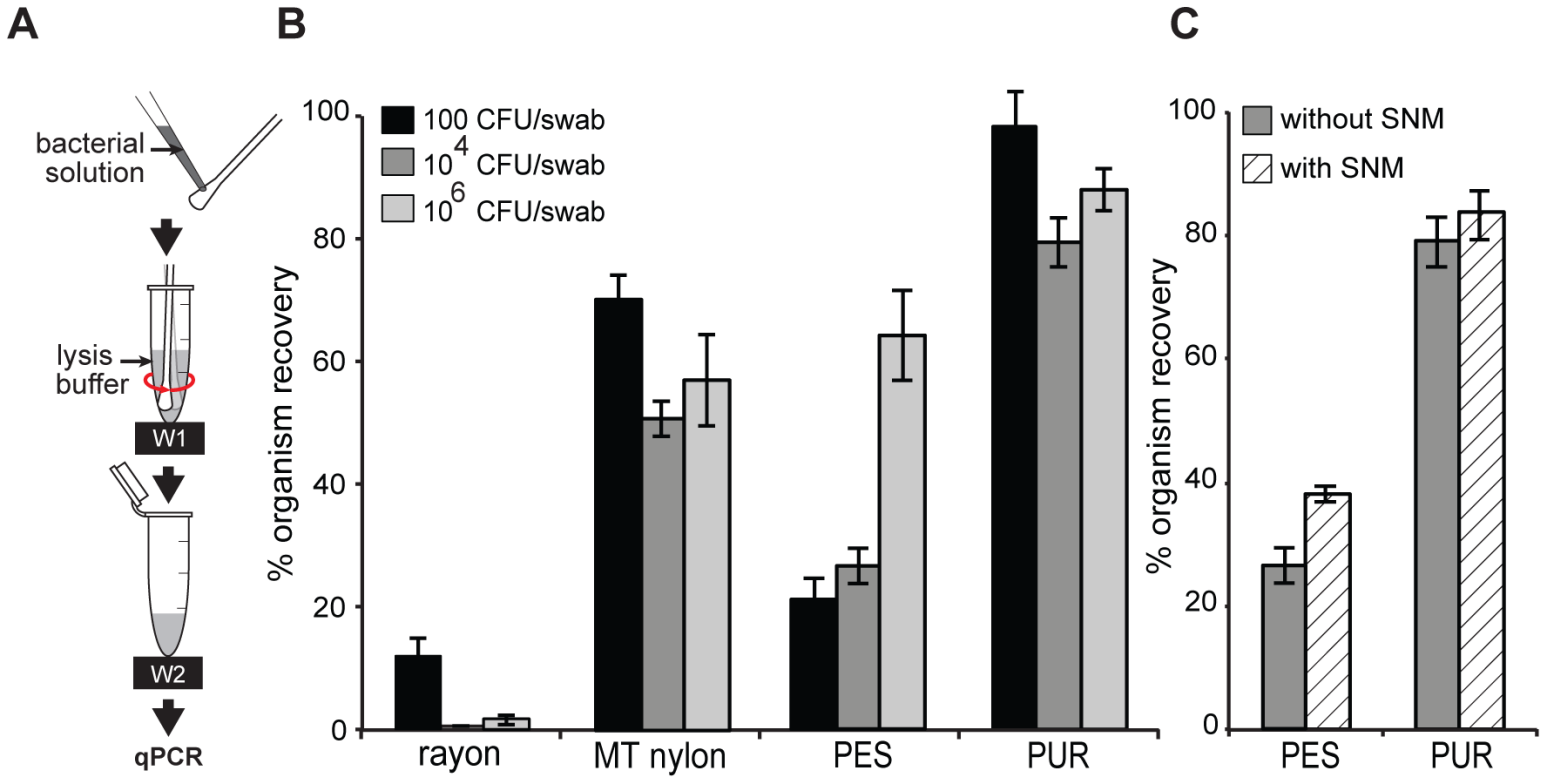
Organism recovery for low-volume samples. (A) Schematic of the experimental set up. 15 µL *S. aureus*/TE (∼100, ∼10^4^, or ∼10^6^ CFU, equivalent to 500, 6×10^4^, or 4×10^6^ *ldh1* copies, respectively, as measured by qPCR) was spiked onto the swab, which was then agitated in 128 µL lysis buffer using 10 second 1 Hz side twirl, and removed. (B) Comparison of the % Organism Recovery in four swabs at three different organism input numbers (mean ± SE, N = 5), which was calculated using Equation 5 in the text. (C) Comparison of the % Organism Recovery (mean ± SE; N = 5) using ∼10^4^ CFU/swab of *S. aureus* in the presence and absence of simulated nasal matrix (SNM).

Swabs were tested with a sample volume that was less than the fluid capacity for all swabs (15 µL). Four dry swab types (PUR foam, knitted PES, rayon, and MT flocked nylon swabs (N = 5)) were tested for organism recovery using low-volume samples (Figure 3A). When bacterial solution was pipetted onto the swab tip, fluid absorption behaved differently among swab types. For PUR swabs, the fluid beaded up on the surface without absorbing. For PES and nylon swabs, the bacterial fluid formed a thin film around swab tip but did not bead up. For rayon swabs, fluid was completely absorbed into swab tips.

The percent organism recovery of each swab using low-volume samples is shown in Figure 3B. Regardless of the number of organisms added, PUR foam yielded the highest organism recovery of 79%–98%. MT nylon and PES had intermediate organism recovery of 51–70% and 21–65%, respectively. Rayon provided the lowest organism recovery of 1–12%. A two-way ANOVA indicated significant effects of swab type and numbers of organisms applied to swab, and a significant interaction (every p<0.05). Post-hoc comparisons showed that organism recovery of PUR was significantly higher than other swabs (followed by MT nylon, PES, and rayon).

Figure 3C compares % organism recovery of PES (N = 5) and PUR (N = 5) for bacterial samples in the presence and absence of simulated nasal matrix (SNM). We verified that SNM did not interfere with qPCR and ACP lysis (Fig. S8). Despite the presence of SNM, PUR swabs still yielded significantly higher organism recovery than PES swabs; mean ± SE was 84±4% for PUR and 38±2% for PES (two-way ANOVA, effect of swab type, p<0.0001). SNM significantly increased % organism recovery (p = 0.03).

#### Excess-volume fluid sample (beyond swab saturation)

**Figure 4:**
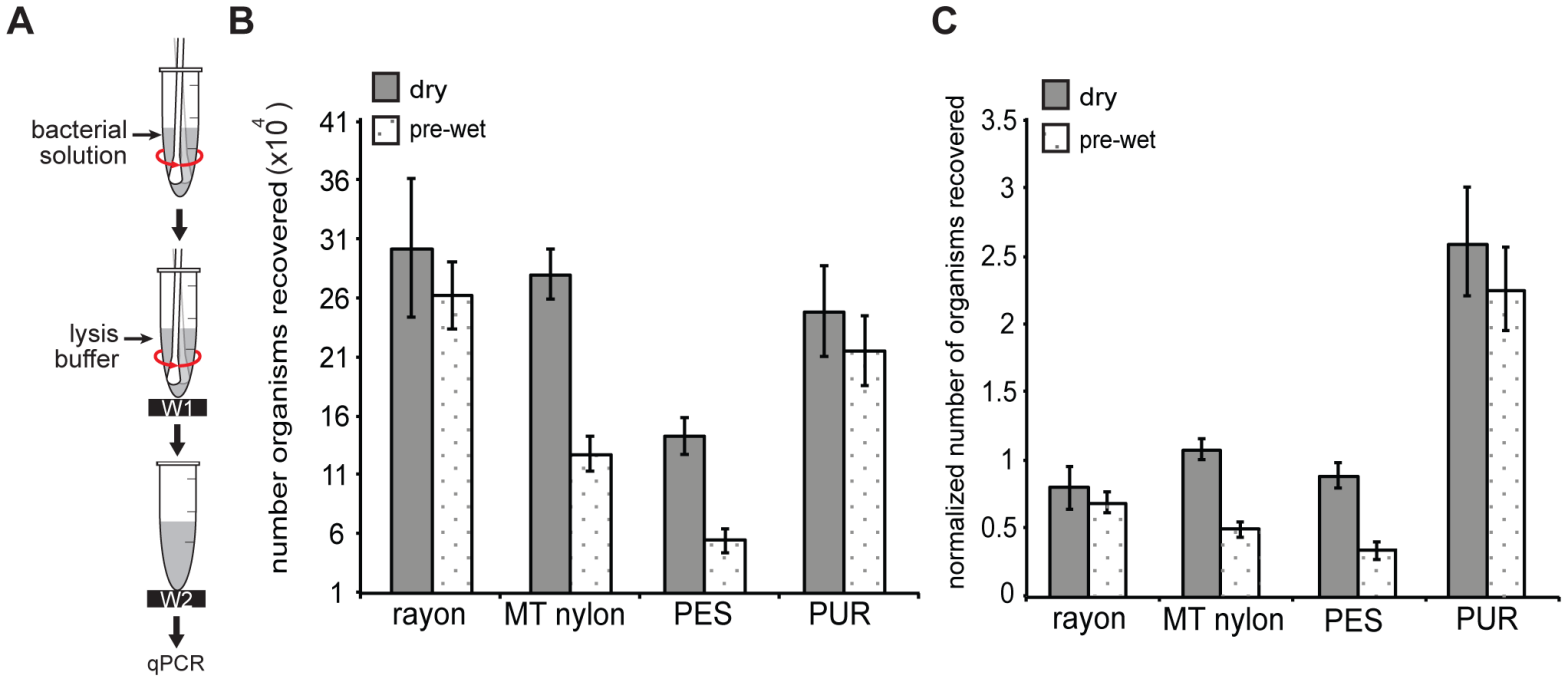
Organism recovery for high-volume samples. (A) Schematic of the experimental set up. Either a dry or pre-wet swab was dipped into 1 mL ∼10^6^ CFU/mL *S. aureus* solution (equivalent to 6×10^6^ *ldh1* copies/mL, as measured by qPCR) and agitated by 10 second 1 Hz side twirl. The swab was then inserted into 128 µL lysis buffer, agitated by 10 second 1 Hz side twirl, and removed. (B) Comparison of the absolute number of organisms recovered for dry and pre-wet swabs. Absolute organism recovery was reported (rather than %) since the uptake of sample volume was different for each swab; absolute recovery was calculated using Equation 4 in the text. In all cases, recovery was larger than would be expected based on swab volume and sample concentration by colony counts due to presence of multiple target copies per CFU. (C) The number of organisms recovered from each swab from panel (B) normalized by the number of organisms expected based solely on the sample concentration and volume capacity of the swab (estimated number of organisms collected by the swab  =  swab volume capacity (µL) x bacterial stock concentration (copies/µL from qPCR)).

Four swabs (rayon, PES, PUR, and MT nylon (N = 5)) were tested using excess-volume samples (1 mL). As the swab was submerged into 1 mL bacterial solution and twirled (Fig. 4A), dry rayon, PUR and PES swabs released air bubbles and allowed solution to flow into the interior of the swab tip. Figure 4B reports the number of organisms released from the swab into lysis buffer. A two-way ANOVA showed a significant effect of swab type on the number of organisms recovered (p = 0.0001) and pre-wetting the swab (p = 0.001). No significant interaction was found (p = 0.63). Post-hoc analysis indicated that PES swabs yielded significantly fewer organisms recovered than rayon, MT nylon and PUR. Wetting the swab reduced number of organisms recovered. The number of organisms recovered from each swab was then normalized by the product of its volume capacity and the concentration of organisms in the bacterial sample solution (Fig. 4C). Two-way ANOVA indicated significant effect of swab type (p<0.0001) and pre-wetting the swab (p = 0.009). Post-hoc analysis reported that PUR swabs had significantly higher normalized organism recovery than other swabs.

#### Sample dried on a surface

**Figure 5:**
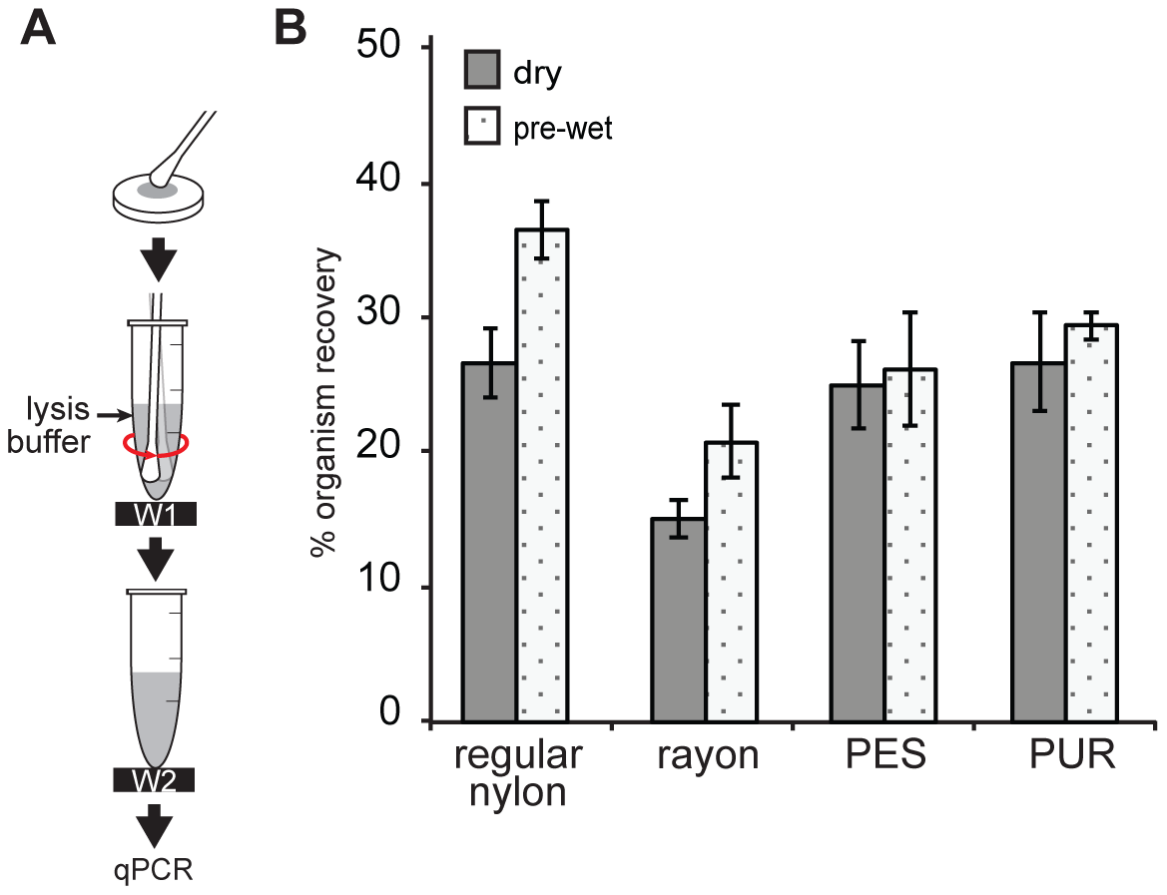
Organism recovery for dried samples. (A) Schematic of the experimental set up. 15 µL of *S. aureus* solution (∼10^4^ CFU, equivalent to 6×10^4^ *ldh1* copies, as measured by qPCR) was spotted on a 25/64-inch diameter PDMS punch and left to dry. A dry or pre-wet swab was rubbed on the PDMS surface (10 times), agitated in 128 µL lysis buffer using 10 second 1 Hz side twirl, and removed. (B) Comparison of % organism recovery for pre-wet and dry swabs based on a control sample and an assumption of 100% collection efficiency.

Four swabs (PUR, PES, rayon, and regular-tip flocked nylon swabs (N = 5)) were tested for organism recovery using a dried bacterial sample (Fig. 5A). Swabs were dry or pre-wet (by dipping into TE buffer). To ensure that a swab was able to pick up the dried sample effectively, the swab was rubbed vigorously on the surface of PDMS (Fig. S2). Figure 5B shows % organism recovery for dried samples. A two-way ANOVA indicated % organism recovery was affected by swab type (p = 0.0004) and wet/dry swab pre-conditions (p = 0.02) with no significant interaction (p = 0.4). Post-hoc comparisons showed rayon had significantly lower % organism recovery than other swabs.

#### Robustness to user variations in manual agitation

**Figure 6:**
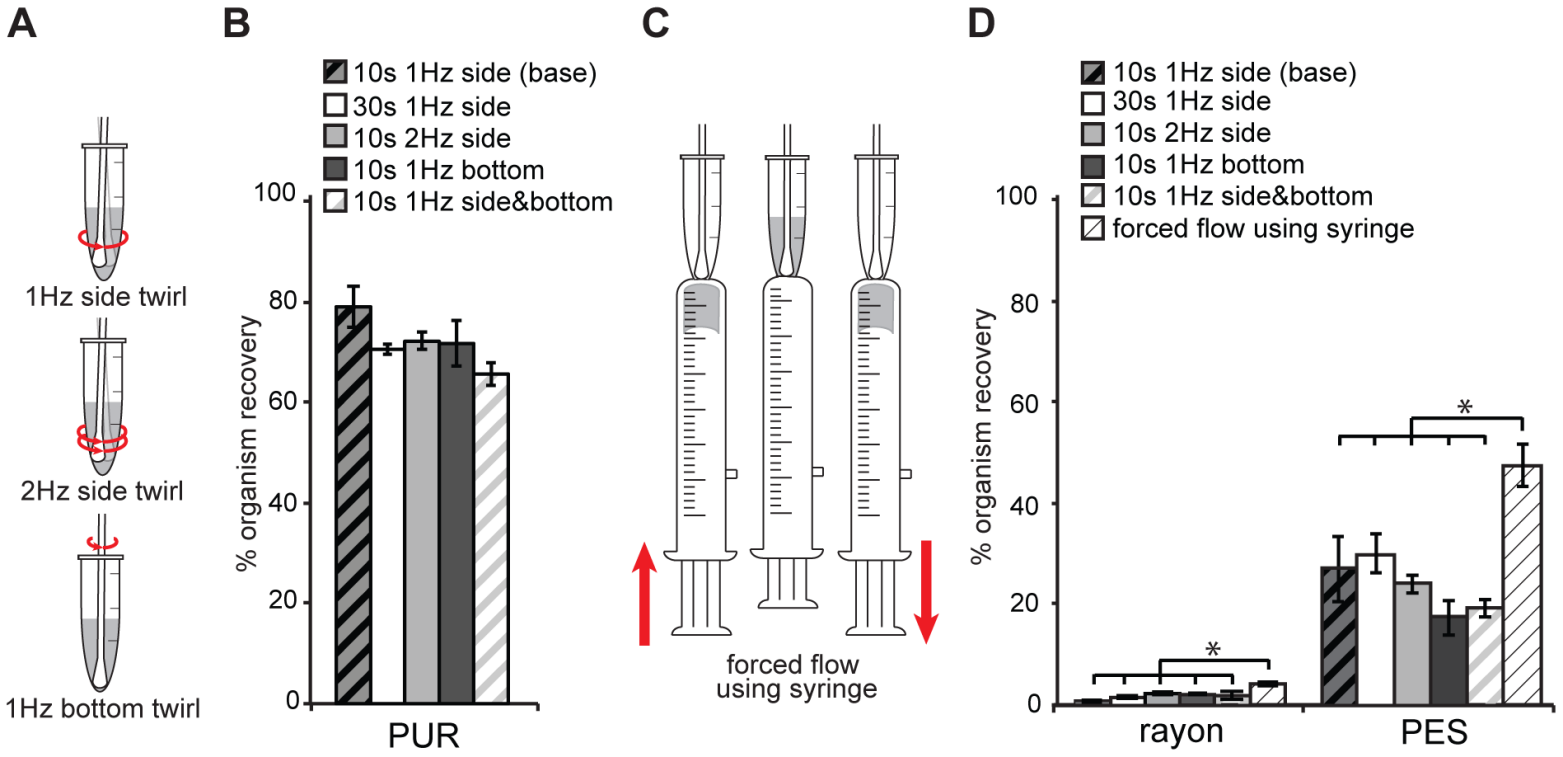
Comparison of manual agitation methods for swab transfer. (A) Schematic of action performed over a period of 1 second for different manual twirling methods. (B) Comparison of % organism recovery of PUR swabs using different twirling methods, which was calculated using Equation 5 in the text. (C) Schematic of the new forced flow syringe method. (D) Comparison of % organism recovery for PES and rayon swabs, using different twirling methods and the forced flow syringe method. * indicates statistically significant differences (Tukey-Kramer, α = 0.05).

Three dry swab types (PUR, PES, and rayon (N = 5)) were tested for variations in manual agitation to release organisms into the lysis buffer (Fig. 6A). All manual twirl methods yielded comparable % organism recovery from PUR swabs (Fig. 6B); the low variation in PUR swabs was likely due to lack of sample absorption into the hydrophobic swab tip, which allowed sample release without agitation. Greater variation was observed in PES and rayon swabs (Fig. 6D), which represented more realistic sampling conditions. Coefficients of variation of organism recovery of all manual twirling methods were 7% for PUR, 23% for PES and 40% for rayon swabs.

#### Engineering for improved recovery

A new manual agitation method, forced fluid flow using a syringe, was developed to increase organism recovery (Fig. 6C). In order to test effectiveness of this method in improving organism recovery, intermediate-performing swabs (rayon and PES; Fig. 3D) were selected. We compared % organism recovery derived from the forced-flow using the syringe method to other agitation methods (Fig. 6D). A one-way ANOVA rejected the hypothesis that there was no difference among the six agitation methods. Post-hoc comparison (Tukey-Kramer, α = 0.05) indicated that the forced flow using the syringe method yielded significantly higher organism recovery than the other five methods. This was true for both swab types.

#### Bench-top gold standard method

Six swabs (PUR, PES, MT nylon, regular-tip nylon, cotton, and rayon) were tested for their organism recovery when 10 seconds of vortexing was applied. Organism recovery of cotton swabs could not be reported, since they absorbed all of the sample fluid. For the rest of swab types, a one-way ANOVA rejected the hypothesis that there was no difference among swab types. Post-hoc comparison (Tukey-Kramer, α = 0.05) indicated that PUR, PES, and MT nylon yielded significantly higher organism recovery than rayon and regular-tip nylon (Fig. S9). In comparing the efficiency of vortexing and 1 Hz side twirl, vortexing offered a significant improvement over 1 Hz side twirl methods for PES, MT nylon, and rayon (p<0.001 in all cases). However, vortexing did not significantly increase organism recovery in PUR (p>0.05). Additionally, vortexing yielded a significantly higher organism recovery for PES and rayon compared to forced flow using syringe method (p>001 in both cases).

## Discussion

In this study, we characterized several important properties of swabs currently utilized world-wide in many POC diagnostic tests. The experimental design had three overall aims: 1) to provide quantitative analysis and definitions of swab transfer efficiency that can be used to evaluate swabs and transfer methods, 2) to identify differences in swab transfer efficiency for a selection of common swabs and sample conditions, and 3) to identify commercially-available swabs that perform well for sample conditions and manual agitation methods typical of POC testing. The results demonstrate that the choice of swab type for POC testing may be critical to achieving a sensitive and reliable test.

We built on previous work to develop analysis methods and definitions for quantitative evaluation of swab transfer efficiency. Some studies have assumed that the volume absorbed is an accurate representation of organisms picked up by the swab [14], [21], [22]. This assumption applies only when organism densities are the same inside and outside the swab; it will be inaccurate if organisms accumulate in the swab during agitation or if organisms do not flow freely into the swab. Our results show that swab transfer efficiency cannot be predicted based on fluid volume recovery alone, rather it requires analysis of organism recovery. The method for measuring the number of organisms in a sample also varies across studies. For bacteria, common measures include optical density at 600 nm (OD_600_), colony counts (as colony forming units, CFU) [14] [23]–[25], and quantitative polymerase chain reaction (qPCR). However, OD is only applicable to high organism density. In colony counting, the transfer procedure itself can affect organism vitality (e.g., due to fluid composition, physical damage by vortexing or agitation) [26]. For clustering or biofilm-forming organisms [27], such as *S. aureus*, fragmentation during the transfer procedure can artificially increase the apparent recovery [26]. Thus, colony counting can bias results when comparing transfer methods or using a control to calculate percent organism recovery since each sample experiences different conditions. In contrast, qPCR provides consistent results independent of cell viability or clustering [28] [29], and it allows calculation of transfer efficiency by normalizing organism recovery to an input control sample analyzed by the same method. Our results demonstrate that qPCR and input controls allow quantitative measurement of swab transfer efficiency that can be applied across studies.

The scenario of low-volume samples showed the largest difference between swab types. For example, the sample beaded on the hydrophobic tip of the PUR foam swab and was promptly released into the lysis buffer; this scenario is not a realistic sampling case since the swab resists collection of the small fluid volume, and the high recovery should be interpreted with caution (although we note that PUR swabs also gave high recovery for high-volume samples and dry samples). In less hydrophobic materials such as PES and MT nylon, a thin film coating of bacterial solution was created surrounding the swab tip resulting in intermediate recovery. The hydrophilic tip of the rayon swab [14] fully absorbed the small sample solution, and absorption of transfer fluid may have further driven sample into the swab interior resulting in poor recovery during agitation. Thus, the wettability of the swab itself may impact specimen recovery.

Intuitively, swabs with larger volume capacity will collect more organisms when excess fluid sample is available. We hypothesized that, in addition to the swab volume capacity, swab chemical composition and structure can impact organism pick up and release. To investigate this, we normalized the number of organisms recovered by the estimated number of organisms that would have been picked up if there were no impact of material composition and structure. The nylon, PES, and rayon swabs all transferred near the expected number of organisms based on their volume capacity, but the normalized organism recovery for the PUR swab was more than 2.5 fold higher than the other swabs (Fig. 4C). This result suggests that swabs may accumulate organisms during agitation. Surprisingly, the PUR and rayon swabs maintained their recovery under pre-wet conditions. For the PUR swab, the large open pore structure may have allowed effective exchange of pre-wetting fluid with sample during collection. For the rayon swab, the large tip and dense structure may have prevented access to sample in the swab interior (as in Figure 3) for both dry and pre-wet conditions; the good recovery would then imply that rayon also accumulated organisms on its outer surfaces during collection. The results suggest that swab composition and structure can have a significant impact on collection and release efficiency and should be evaluated for the specific organism and sample type.

Swabs can be used to collect samples from dry surfaces, such as for environmental testing and sampling from skin or dry nasal passages, and pre-wet swabs are sometimes used to increase collection efficiency for dry samples [24]. The improved performance of rayon swabs with dry samples (Fig. 5B) compared to the low-volume case (Fig. 3B) suggests that organisms collected from a dry surface were more accessible to manual agitation. This would be expected since dry collection deposits sample on the exterior surface of the swab, whereas liquid samples wick into the swab interior where they may be inaccessible by manual agitation. Similarly, the effect of pre-wetting was more pronounced for rayon and regular nylon (Fig. 5B), which have relatively high volume absorption compared to PES and PUR (Fig. 2B). The pre-wetting step filled the interior volume of swabs prior to sampling, and presumably this allowed dried sample to remain on the exterior surface of swabs where they were more effectively released during manual agitation.

Biological fluids found in clinical specimens can affect organism recovery. Complex matrices can affect physical properties (e.g., viscosity) or chemical properties (e.g., binding to swab materials or passivating swab surfaces). For example, mucin has been found to reduce non-specific binding of protein and has been used to coat biomaterials to create non-fouling surfaces [30] and repel other negatively charged molecules [31] (e.g., DNA). However, the increased viscosity of complex matrices may reduce the effectiveness of agitation. As an example of a sample matrix, we measured recovery in the presence of simulated nasal matrix (SNM) and found that it had little effect on organism recovery (Fig. 3C). The effect of sample matrix will be highly dependent on the sample type and should be evaluated for each application; the experimental design used here for SNM can be applied to quantitatively measure the effects of sample matrix on swab transfer efficiency.

User variations in swab transfer procedure could affect test sensitivity and reproducibility. Commercial LFTs typically include instructions to agitate the swab for a given time but do not specify the method. Our results with selected swabs showed that variations in agitation method and time had modest impact on organism recovery for the case of low-volume samples. Robustness to user variation will be especially important to maintain sensitivity and reproducibility for POC tests performed by untrained users and should be tested for all applications.

We demonstrated that an engineered swab transfer method can increase recovery from poorly performing swabs. However, the improved organism recovery was still significantly lower compared to vortexing (gold standard). Other engineered methods could include buffer-filled swab shafts that push fluid from the swab interior or methods that compress the swab to remove absorbed fluid. This finding has direct application to future developments in POC testing.

The PUR swab was the best performing swab across all sample conditions. This may be of clinical interest as similar PUR swabs have been found to be sensitive for the detection of respiratory viruses in immunocompetent and immunocompromised human subjects [32]. In addition, smaller swab size may be applicable to clinical use in subjects of diverse size and in sampling of diverse structure. The small volume of the PUR swab and reproducible recovery for variations in agitation methods also make it well-suited for application in POC devices.

Limitations of this work included the use of analytical samples instead of clinical samples, and the use of a single organism, *Staphyloccocus aureus*, as a model due to its relevance as a human pathogen. However, these choices allowed us to create replicable cultured samples with known numbers of organisms and to create various sample types representative of swab collection sites (from dry to wet samples, in the presence or absence of other biological components). Other limitations of this work include the evaluation of a limited number of swab types, although they represent some of the most common commercially-available swab types used in clinical testing today. Finally, although the number of organisms transferred from swabs involves both collection and release, swab collection efficiency is highly dependent on the target pathogen and details of the sampling site; our work only focused on swab transfer release efficiency.

## Conclusions

We have built on previous work to develop a quantitative method to evaluate and compare swab transfer performance. We evaluated a variety of swabs under manual agitation conditions appropriate for POC testing. By selecting a set of commercially-available swabs representing a variety of tip sizes, shapes, and materials, and utilizing qPCR as a direct measure of target quantity, we were able to quantitatively measure the transfer efficiency of a model organism. Our data show that swab size, structure, or composition affects swab release performance under different sampling conditions (low-volume, excess volume, or dry samples). Variations in manual agitation method and time had modest impact on swab transfer efficiency for three swabs tested, which is encouraging considering the likelihood of user variation in POC tests. For cases when a test is constrained to a swab with poor transfer efficiency, we demonstrated how forced-flow transfer methods could be used to improve transfer efficiency. The results and discussion presented here highlight key factors that should be considered in selecting swabs for POC applications. The quantitative evaluation methods developed here can be applied to other swab types in the future, both for POC applications or laboratory tests.

## Supporting Information

Figure S1 **Sensitivity of the MRSA/SA ELITe MGB qPCR assay. 10-fold serial dilutions of** ***ldh1*** **copies (5 to 5×10^5^ copies/reaction) and in negative TE control (N = 3) were analyzed by the qPCR assay.** The result shows the linearity across the concentration range. Five copies/reaction (∼1 CFU/reaction) was detected reproducibly (mean ± SE  = 5.08±0.314, CV  = 0.107) while none of the negative TE controls was falsely detected.
(EPS)

Figure S2 **Schematic of how swab was rubbed on PDMS surface.** Bacterial sample was spotted on the PDMS punch and dried. Starting at step 1, the swab was rubbed on the surface, from left to right. Then, the shaft was rotated 45 degrees clockwise in step 2. After that, the swab was moved to the left position to complete the cycle in step 3. In each test case, 10 cycles of 3 steps were conducted.
(EPS)

Figure S3 **Scanning electron micrographs of swab tips:** (A) mid-turbinate nylon (B) polyurethane (C) polyester (D) rayon. Scanning electron micrographs were obtained using an FEI Sirion scanning electron microscope. Samples were sputtered with an 11 nm Au/Pd coating prior to imaging (SPI Module Control, Structure Probe, Inc., West Chester, PA, USA.) SEM imaging and sputter coating work was performed at the University of Washington Nanotech User Facility (NTUF), a member of the NSF-sponsored National Nanotechnology Infrastructure Network (NNIN).
(EPS)

Figure S4 **Volume recovery testing of calcium alginate (dissolvable) swab.** TE buffer was spiked with Allura red in order to aid visualization of 1% w/v sodium citrate buffer (intended to dissolve swab tip material). (A) After 10 second 1 Hz twirl in 128 µL citrate buffer, swab absorbed the majority of eluate into its tip, and fluid around the tip turned into a glue-like consistency (in panel A, the tube is suspended by the swab). (B) Repeated experiment with an attempt to dissolve swab tip material in 1 mL sodium citrate. After 1 min 1 Hz side twirl, (C1) swab tip could not be fully dissolved. After swab removal, (C2) eluate appeared viscous and stuck to the bottom of the tube against gravity.
(EPS)

Figure S5 **ACP lysis efficiency at various treatment times and temperatures.** Our ACP lysis method was adapted from the method previously reported by Patel *et al* [20]. The lysis time and temperature was changed from 15 minutes at 37°C to 2 minutes at room temperature to be more appropriate for low-resource settings. We observed no growth when plating the bacterial lysate on a tryptic soy agar (data not shown), which confirmed 100% killing efficiency. Furthermore, we compared the amount of amplifiable DNA for different times and temperatures. 2.5×10^5^ CFU in 100 µL *S.aureus*/TE solution with an addition of 17.6 µL ACP (20 U/µL) underwent ACP lysis for 2, 5, 15, 25 minutes at room temperature and 37°C (N = 3). Then, the ACP was deactivated for 10 minutes at 95°C. 82.4 µL of TE was added to bring up the final volume of lysate to 200 µL. 2 µL lysate was amplified using *ldh1* qPCR assay (one *S.aureus* organism has one copy of the *ldh1* gene) to estimate amplifiable DNA (copies/reaction). All tested conditions yielded relatively high *ldh1* copies/reaction and there was no significance difference among cases (two-way ANOVA, p = 0.1912 for time variables and p = 0.0943 for temperature variables). Based on the ACP lysis (2 minutes at room temperature), estimated number of *ldh*1 copies/CFU  = 6.4, (16000 copies/2 µL lysate):(2.5×10^5^ CFU/200 µL lysate). Note that the vertical axis does not span to zero, which makes error bars appear artificially large.
(EPS)

Figure S6 **Testing bacteria and bacterial DNA contamination in swabs and other materials.** The eluate from different dry swabs, PDMS punches, and forced-flow syringes was tested for bacteria and bacterial DNA contamination. (A) Swabs (PES, PUR, MT Nylon, Regular Nylon, Rayon (N = 3)) were dipped into 128 µL TE buffer and agitated (10 seconds, 1 Hz twirl) to generate eluate. (B) PDMS punches (N = 3) were added to 128 µL of TE buffer and vortexed for 10 seconds to generate eluate. (C) Forced-flow syringes (N = 3) were used to draw 128 µL TE buffer; the fluid was introduced to the tube adhered on the top of the syringe and purged via the side outlet to generate eluate. (D) Swabs were wiped onto the TSA plate. 30 µL of the eluate from PDMS punches and forced-flow syringe were spread onto the TSA plate. The plates were cultured overnight at 37°C to observe the growth of bacteria. None of plates with the eluates from swabs and other materials was devoid of bacterial colonies, except the positive control (30 µL of 10^5^ CFU/mL *S.aureus*). This demonstrated that the swabs and all materials were not contaminated with bacteria. (E) Positive controls (10-fold serial dilution of 50 to 5×10^5^ *ldh1* copies) and negative controls (9 µL of eluate from swabs, punches, and syringes) were analyzed by the qPCR assay. The qPCR did not detect any *ldh1* copies in eluate. This confirmed the absence of bacterial DNA contamination.
(EPS)

Figure S7 **Testing effect of swab components on ACP lysis and qPCR.** The eluate from different dry swabs was added to the qPCR reaction or ACP lysis mixture to test for chemical interference by components leached from the swabs. Swabs (PES, PUR, MT Nylon, Rayon, Cotton (N = 3)) were dipped into 128 µL TE buffer and agitated by 10 second 1 Hz twirl to generate swab eluate. Lysis and qPCR efficiency in presence and absence of swab eluate from each swab type were compared. (A) For qPCR experiments, each reaction contained the mixture of 5 µL of 10,000 *S. aureus* genomic copies with 4 µL of different swab eluate or 4 µL of TE alone. *ldh1* copies (mean ± SE) estimated by qPCR were plotted. (B) For lysis experiments, the mixture of 50 µL of 5×10^4^ CFU of *S. aureus/TE* and 50 µL of different swab eluate or TE alone were mixed with 17.6 ACP (20 U/µL) and underwent lysis at room temperature for 2 minutes. The ACP was then deactivated by heating at 95°C for 10 minutes. 9 µL of lysate was analyzed by qPCR. *ldh1* copies/reaction in the lysate (mean ± SE) of each sample was plotted. Based on the ACP lysis (in TE case) the estimated number of *ldh1* copies/CFU was 6.5: (25,000 copies/9 µL lysate):(5×10^4^ CFU/117.6 µL lysate). No significant difference among samples was found in A and B (One-way ANOVA, p = 0.4211 and 0.7073 respectively). Note that the vertical axis does not span to zero, which makes error bars appear artificially large.
(EPS)

Figure S8 **Effect of simulated nasal matrix (SNM) on qPCR and ACP lysis efficiency.** 100 µL of 10^5^ CFU *S. aureus* solution/TE, either with or without the addition of 15 µL of SNM, was mixed with 17.6 µL of ACP (20 U/µL) and then kept at room temperature for 2 minutes. The lysis was then deactivated by heating at 95°C for 10 minutes and each tube was added with TE to bring up the final volume to 200 µL. 2 µL of final lysate was added in each qPCR reaction. The graph compares the number of *ldh1* copies/reaction (mean ± SE; N = 4) from lysate in both cases (with and without SNM). Based on the lysate without SNM, the number of *ldh1* copies/CFU was 3; (3,000 copies/2 µL lysate):(10^5^ CFU/200 µL lysate). One-way ANOVA did not indicate the significant difference (p = 0.08). Note that the vertical axis does not span to zero, which makes error bars appear artificially large.
(EPS)

Figure S9 **Vortexing (gold standard) swab transfer.** (A) Schematic of experimental set up. 15 µL *S. aureus*/TE (10^4^ CFU equivalent to 6×10^4^ *ldh1* copies) was spiked onto the swab, which was then agitated in 128 µL lysis buffer using 10-second vortexing, and removed. (B) Comparison of the % Organism Recovery in five swabs (mean ± SE, N = 5), which was calculated using Equation 5 in the text. (C) Comparison of the % Organism Recovery of four swabs (mean ± SE; N = 5) using vortexing and manual 10 second, 1 Hz side twirl. (D) Comparison of the % Organism Recovery of two swabs (mean ± SE; N = 5) using vortexing and forced flow using syringe method.
(EPS)

Table S1 **Summary of experimental data (mean ± SE, N = 5).**
(EPS)

Table S2 **Coefficient of variation (CV) of experimental data (N = 5).**
(EPS)
